# High prevalence of multidrug resistant ESBL- and plasmid mediated AmpC-producing clinical isolates of *Escherichia coli* at Maputo Central Hospital, Mozambique

**DOI:** 10.1186/s12879-020-05696-y

**Published:** 2021-01-06

**Authors:** Calvina E. L. Estaleva, Tomas F. Zimba, John Osei Sekyere, Usha Govinden, Hafizah Y. Chenia, Gunnar S. Simonsen, Bjørg Haldorsen, Sabiha Y. Essack, Arnfinn Sundsfjord

**Affiliations:** 1 Microbiology Laboratory, Maputo Central Hospital, Maputo, Mozambique; 2 High Institute of Health Sciences (ISCISA), Maputo, Mozambique; 3 Antimicrobial Research Unit, School of Health Sciences, University of KwaZulu-Natal, Durban, South Africa; 4 Discipline of Microbiology, School of Life Sciences, University of KwaZulu-Natal, Durban, South Africa; 5 Department of Microbiology and Infection Control, Norwegian National Advisory Unit on Detection of Antimicrobial Resistance, University Hospital of North Norway, Tromsø, Norway; 6 Research Group for Host-Microbe Interaction, Department of Medical Biology, Faculty of Health Sciences, UiT Arctic University of Norway, NO-9037 Tromsø, Norway

**Keywords:** *Escherichia coli*, Multidrug resistance, Extended-spectrum β-lactamase, Plasmid-mediated AmpC β-lactamase

## Abstract

**Background:**

Epidemiological data of cephalosporin-resistant *Enterobacterales* in Sub-Saharan Africa is still restricted, and in particular in Mozambique. The aim of this study was to detect and characterize extended-spectrum β-lactamase (ESBL) - and plasmid-mediated AmpC (pAmpC)-producing clinical strains of *Escherichia coli* at Maputo Central Hospital (MCH), a 1000-bed reference hospital in Maputo, Mozambique.

**Methods:**

A total of 230 clinical isolates of *E. coli* from urine (*n* = 199) and blood cultures (*n* = 31) were collected at MCH during August–November 2015. Antimicrobial susceptibility testing was performed by the disc diffusion method and interpreted according to EUCAST guidelines. Isolates with reduced susceptibility to 3rd generation cephalosporins were examined further; phenotypically for an ESBL−/AmpC-phenotype by combined disc methods and genetically for ESBL- and pAmpC-encoding genes by PCR and partial amplicon sequencing as well as genetic relatedness by ERIC-PCR.

**Results:**

A total of 75 isolates with reduced susceptibility to cefotaxime and/or ceftazidime (*n* = 75) from urine (*n* = 58/199; 29%) and blood (*n* = 17/31; 55%) were detected. All 75 isolates were phenotypically ESBL-positive and 25/75 (33%) of those also expressed an AmpC-phenotype. ESBL-PCR and amplicon sequencing revealed a majority of *bla*_CTX-M_ (*n* = 58/75; 77%) dominated by *bla*_CTX-M-15_. All AmpC-phenotype positive isolates (*n* = 25/75; 33%) scored positive for one or more pAmpC-genes dominated by *bla*_MOX/FOX_. Multidrug resistance (resistance ≥ three antibiotic classes) was observed in all the 75 ESBL-positive isolates dominated by resistance to trimethoprim-sulfamethoxazole, ciprofloxacin and gentamicin. ERIC-PCR revealed genetic diversity among strains with minor clusters indicating intra-hospital spread.

**Conclusion:**

We have observed a high prevalence of MDR pAmpC- and/or ESBL-producing clinical *E. coli* isolates with FOX/MOX and CTX-Ms as the major β-lactamase types, respectively. ERIC-PCR analyses revealed genetic diversity and some clusters indicating within-hospital spread. The overall findings strongly support the urgent need for accurate and rapid diagnostic services to guide antibiotic treatment and improved infection control measures.

**Supplementary Information:**

The online version contains supplementary material available at 10.1186/s12879-020-05696-y.

## Background

The World Health Organization recognizes antimicrobial resistance (AMR) as a major global public health problem [1, 2]. The dissemination of multidrug-resistant (MDR), extended-spectrum β-lactamase (ESBL)-producing *Enterobacterales* is of particular concern limiting treatment options for invasive infections to last line antibiotics [3–5].

ESBLs are enzymes that hydrolyze an extended spectrum of β-lactam antibiotics including penicillins and oxyimino-cephalosporins, but not cephamycins [6]. The most widely distributed ESBL enzymes are CTX-M-type β-lactamases, which preferentially hydrolyze cefotaxime. The worldwide dissemination of CTX-M-type β-lactamases has been dramatic and greater than the impact of the TEM- and SHV-type ESBLs [5, 7].

Plasmid-mediated AmpC β-lactamases (pAmpCs) are also an important cause of broad spectrum β-lactam resistance in Gram-negative bacteria [8]. Many Gram-negative bacteria including most *Enterobacterales* have chromosomally-encoded AmpC-β-lactamases. Some AmpC-genes have been mobilized from their chromosomal origin and may be spread by plasmids (pAmpC), with *bla*_MOX/FOX/DHA/CMY_ being the most prevalent variants [6, 8, 9]. In general, AmpC β-lactamases confer resistance to a wide range of β-lactam drugs including penicillins, cephamycin, 1st – 3rd generation cephalosporins as well as classical β-lactamase inhibitors like clavulanic acid and tazobactam to which ESBLs are sensitive [6]. Co-presence of ESBLs and pAmpCs may occur [10].

In the context of low- and medium income countries (LMICs), and sub-Saharan Africa in particular, there are knowledge gaps concerning the epidemiology of ESBL- and pAmpC-producing *Enterobacterales* in different ecological settings [11–13]. This study delineates plasmid AmpC- and ESBL-mediated resistance in clinical isolates of *E. coli* processed at the Maputo Central Hospital, Mozambique, in 2015.

## Methods

### Bacterial isolates

The strain collection consisted of isolates recovered from clinical specimens referred to the Department of Clinical Microbiology, Maputo Central Hospital, a 1000-bed reference hospital in Maputo, Mozambique. We included urine and blood culture samples from mid-August through mid-November 2015. Clinical information was obtained from the laboratory request form only, allowing distinction between in- and outpatients. Urine samples were obtained from both in- and outpatient whereas blood cultures were from inpatients only. Only one isolate per patient was included in the study.

### Bacterial species identification and Antimicrobial Susceptibility Testing (AST)

*E. coli* strains were identified by standard biochemical tests including the API-20E kit (bioMerieux, la Balme-les-Grottes, France). AST was performed using the disk diffusion method according to EUCAST [14] and corresponding 2015-guidelines for interpretation including ampicillin (10 μg), amoxicillin-clavulanic acid (20–10 μg), piperacillin-tazobactam (30–6 μg), cefoxitin (30 μg), cefotaxime (5 μg), ceftazidime (10 μg), ceftriaxone (30 μg), meropenem (10 μg), ciprofloxacin (5 μg), gentamicin (10 μg), trimethoprim-sulfamethoxazole (1.25–23.75 μg), and nitrofurantoin (100 μg). MDR was defined as acquired resistance to at least one agent in three or more antimicrobial classes [15].

Quality assurance was performed weekly during the study period using the wild-type *E. coli* ATCC 25922 strain. Moreover, *K. pneumoniae* ATCC 700603 (ESBL-type *bla*_SHV-18_) and *E.coli* A5–8 (pAmpC-type *bla*_DHA_) were used as ESBL- and AmpC-positive controls, respectively. *E. coli* A5–8 is a well characterized strain that was obtained from the Norwegian Advisory Unit for Detection of Antimicrobial Resistance. Strains were stored in skimmed milk broth with 10% glycerol at − 70 °C until molecular analysis.

### Phenotypic detection of β- lactamases

Isolates that showed reduced susceptibility (I or R) to cefotaxime and/or ceftazidime were examined for ESBL-production. Moreover, isolates with reduced susceptibility to cefotaxime and/or ceftazidime and cefoxitin were examined for increased AmpC-production [16]. The mechanisms of resistance were examined using ROSCO combined disc tablets (Rosco Diagnostic, Denmark) for ESBL and/or increased AmpC-production and interpreted according to manufacturer’s instructions [17].

### Genetic detection and characterization of β-lactamases

Genetic characterization was performed at the Antimicrobial Research Unit, University of KwaZulu-Natal (UKZN). Phenotypical ESBL-positive isolates were examined for the presence of *bla*_TEM_, *bla*_SHV_, *bla*_CTX-M_, *bla*_CMY_, *bla*_DHA_, *bla*_FOX_ and *bla*_MOX_ genes using consensus PCRs (Additional file 1 Table S1) as described [18–22]. Briefly, DNA extractions were performed from overnight bacterial cultures using GeneJet Purification kit (Thermo Scientific, USA). The extracted DNA was stored at − 20 °C until use. PCR products were visualized by agarose gel electrophoresis using UV light after staining with ethidium bromide. *Bla*_CTX-M_ amplicons were sent for DNA sequencing at Inqaba Biotec (South Africa).

### Genetic typing of ESBL-producing strains by ERIC-PCR

The clonal relatedness of ESBL-positive isolates was examined by ERIC- PCR and analysed as described [22, 23]. Briefly, PCR products were visualized by agarose gel electrophoresis and genetic variation was analysed using the GelComparII version 6.0 software package (Applied Maths, Belgium) and Unweighted Pair Group Method with Arithmetic Mean (UPGMA) cluster analysis to produce a dendrogram.

## Results

### Bacterial isolate selection, identification and AST

*E. coli* isolates were recovered from a total number of urinary (*n* = 199/823; 24.1%) and blood (*n* = 31/255; 12.1%) culture samples. Urinary tract isolates were from in- (*n* = 109) and out-patients (*n* = 90). All blood culture isolates were from inpatients. A total of 58/199 (29%) of urine isolates (51/109 (47%) and 7/90 (8%) isolates from in- and outpatients, respectively showed reduced susceptibility (R or I) to cefotaxime and/or ceftazidime and as compared to 17/31 (55%) of blood isolates (Additional file 1: Tables S2 and S3). The ESBL confirmation test was positive for all 75/230 (32.6%) isolates tested. The ESBL-positive rates were significantly different in urine isolates from out- versus inpatients, respectively 7/90 (7.7%) and 51/109 (46.8%). Reduced susceptibility to meropenem was not observed. MDR was observed in all ESBL-positive isolates.

Reduced susceptibility to cefoxitin was detected in 41/75 (54.6%) of the isolates tested and further examined for increased AmpC-production; 7/17 and 34/58 were blood and urine isolates, respectively (Tables S2 and S3). A total of 25/41 (60.9%) isolates were confirmed positive for increased AmpC-production using the ROSCO combined disc method, representing 25/75 (33%) of the total ESBL-positive population.

The presence of co-resistance to clinically important antibiotics was also examined (Tables S2 and S3) in isolates from blood: ciprofloxacin (*n* = 12/17: 71%), gentamicin (*n* = 8/17: 47%) and trimethoprim-sulfamethoxazole (*n* = 17/17; 100%), and urine: ciprofloxacin (*n* = 40/58; 69%), gentamicin (*n* = 27/58; 47%) and trimethoprim-sulfamethoxazole (*n* = 55/58; 95%). Interestingly, a majority of the isolates, even pAmpC-negative, expressed resistance to β-lactamase inhibitors (amoxicillin-clavulanic acid and piperacillin-tazobactam) indicating additional resistance mechanisms.

### Detection of genes encoding ESBL and plasmid-mediated AmpC

A total of 58/75 (77%), 39/75 (52%), and 1/75 (1%) ESBL-producing isolates were positive for *bla*_CTX-M_ (Additional file 1: Fig. S1), *bla*_SHV_ and *bla*_TEM_ by PCR, respectively. The *bla*_CTX-M_ amplicons were sequence-typed with the following results: CTX-M-15 (*n* = 24; CTX-M group 1), CTX-M-28 (*n* = 2; CTX-M group 1), CTX-M-117 (*n* = 1; CTX-M group 1), CTX-M-36 (*n* = 1; CTX-M-group 1), CTX-M-164 (*n* = 1; CTX-M group 1) and CTX-M no type (*n* = 29) (Fig. S1). Available resources did not allow sequence typing of SHV-amplicons, but 13/17 (76.5%) of *bla*_CTX-M_-negative, ESBL-producing isolates were *bla*_SHV_-positive, indicating a SHV-ESBL-type. The only *bla*_TEM_ positive isolate was positive for *bla*_CTX-M-15_. A total of 25/75 (33%) isolates examined scored positive for one or more plasmid mediated AmpC-genes; *bla*_CMY_ (*n* = 1), *bla*_DHA_ (*n* = 13), *bla*_MOX_ (*n* = 22), and *bla*_FOX_ (*n* = 24). The isolates contained two (*n* = 15) or three (*n* = 10) pAmpC genes: *bla*_CMY;DHA_ (*n* = 1), *bla*_DHA;FOX_ (*n* = 2), *bla*_MOX;FOX_ (*n* = 12) and *bla*_DHA;MOX;FOX_ (*n* = 10). Importantly, only phenotypical AmpC-isolates were positive for *bla*AmpC.

### ERIC – PCR results

Distinct fragment length polymorphisms were obtained for the 75 isolates tested and used as a means of differentiating *E. coli* isolates (Fig. S1). The absence or presence of a band was also examined in deciding divergence in ERIC-PCR profiles. The profiles comprised between 4 and 12 individual bands, varying in size from 0.5–10 kb (Fig. S1). Visual analysis of the profiles included primary, secondary and tertiary amplification representing different amplification intensities. Primary amplification products refer to fragments of highest intensity on the gels. Secondary amplification products were fragments that are not as intense as the primary amplification products but stronger than the tertiary amplification products, which were the minor amplification products of low intensity. All isolates were typeable and band patterns were reproducible on repeat amplification.

The ERIC-PCR profiles allowed separation of the 75 isolates into 50 types grouped into eight main clusters (A-H), and further sub-divided into two or three main sub-clusters (Fig. S1). The urine isolates were diverse and distributed in all eight identified clusters (clusters A-H) with characteristics as outlined below. Overall, isolates with similar profiles demonstrated different β-lactamase gene content with regard to *bla*_CTX-M_-allelic profile (Fig. S1). However, strains in cluster B (*n* = 12) including nine strains from patients at the pediatric department showed a CTX-M group 1/− 15 profile. *Bla*_SHV-_ (*n* = 39), and/or *bla*pAmpC-positive (*n* = 25) strains were distributed across clusters without any apparent patterns (data not shown). However, *bla*pAmpCs were detected across seven out of eight clusters (A-G) and only present in strains from inpatients, indicating in-hospital plasmid spread between strains. The blood isolates were more clonal in nature and mostly observed in clusters F and G, but predominantly in cluster G. Although closely related, they demonstrated differences in their β-lactamase resistance gene content (data not shown).

## Discussion

Literature reviews show that most studies on the presence of clinical isolates of ESBL-producing *Enterobacterales* in Africa have been conducted in Northern and Eastern Africa, with a relative lack of data in Sub-Saharan Africa [13, 24–26]. In general, and particularly in Mozambique there is very limited data from surveillance or clinical studies documenting the susceptibility pattern of common pathogens. Previous studies have revealed a great variety in proportions of ESBL-producing *Enterobacterales*, underlining the importance of surveillance studies and local data in order to guide antimicrobial therapy and infection control [24, 25].

In this study, the overall prevalence of plasmid-mediated AmpC- and/or ESBL-production in clinical isolates of *E. coli* was 10.8% (25/230) and 32.6% (75/230), respectively. Both phenotypes were present in urinary tract and blood culture isolates and with a significant higher prevalence of ESBL in urinary isolates obtained from inpatients (8%) compared to outpatients (47%). CTX-Ms were the most dominant ESBL-type, with CTX-M group 1 and *bla*_CTX-M-15_ as the major subtype and allele, respectively. Most of the *bla*_CTX-M_ negative, ESBL-positive isolates were negative for *bla*_TEM_, but positive for *bla*_SHV_, indicating an SHV-ESBL-type. This is in accordance with the international situation [5, 7] and the recent meta-analysis of ESBL-producing *Enterobacterales* in East Africa hospitals [24].

Moreover, a study of ESBL-producing *Enterobacterales* in stool samples in Mali, Niger and Cameroon showed that CTX-M was the dominant ESBL-type [26]. Finally, our results are also in line with the recent detection of a relatively high prevalence of ESBL CTX-M-type producing *Enterobacterales* in stool samples from Mozambique university students [22].

A total of 11% (25/230) of the isolates expressed an AmpC phenotype and all those were pAmpC-PCR positive. Surprisingly, all isolates contained two to three pAmpC genes of which *bla*_FOX_ was the most prevalent. These observations are in contrast with the worldwide observations of *bla*_CMY_ as the most prevalent pAmpC gene in *E. coli* populations [8, 9]. CMY-2 has the broadest geographic spread among pAmpCs and is an important cause of extended-spectrum beta-lactam resistance in *E. coli* as well as in non-typhoid *Salmonella* strains in many countries [27]. The finding of multiple pAmpC-*bla* genes in single strains has recently been reported in a Tunisian study. Briefly, a total of 11 out of 75 pAmpC positive clinical strains of *Enterobacterales* were shown to contain up to three different pAmpCs [10]. In contrast to our study, CMY-2 was the most common pAmpC-type in their study. Moreover, the combination of MOX-, FOX- and CMY-2 type enzymes was dominant in their isolates, in contrast to ours that mostly contained MOX- and FOX-types in combination with DHA.

ERIC-PCR has been a useful rapid method in various molecular epidemiological studies to describe the genetic relatedness in *Enterobacterales* strain collections [28]. Our ERIC-PCR results revealed an overall genetic diversity of pAmpC - and/or ESBL -positive *E. coli* strains at the Maputo Central Hospital. The results indicate that there is not a dominant clone of ESBL−/pAmpC positive *E. coli*. However, there are several clusters with clonal relatedness indicating minor outbreaks between patients at specific departments. This notion is supported by the isolation of CTX-M-15 producing strains with similar resistance patterns from the Pediatric department linked in time within cluster B.

The observation of multidrug-resistant pAmpC- and/or ESBL-producing *E. coli* in a high proportion of clinical isolates during a period of 3 months is a major concern. *E. coli* is the most prevalent cause of urinary tract infections and Gram-negative bacteremia in most countries [29, 30]. A large proportion of the ESBL-producing strains also expressed resistance to fluoroquinolones, aminoglycosides and trimethoprim-sulfamethoxazole, limiting treatment options to last resort antibiotics such as carbapenems, piperacillin-tazobactam, colistin or tigecycline. Those drugs are not easily available at Maputo Central Hospital and in developing countries in general. Recent 2015-data from the Pharmacy Department at Maputo Central Hospital showed that betalactams represented 75% of the total in-house antibiotic use, in which ceftriaxone (a third generation cephalosporin) covered 21% of the betalactams (Zimba TF et al. unpublished). Carbapenems were not in use in 2015. Thus, a significant proportion of clinical *E. coli* strains at the Maputo Central Hospital is in fact not treatable with the locally available antibiotics. This is in line with the recently reported antimicrobial surveillance data of blood culture Gram-negative pathogens from Blantyre, Malawi [31].

In conclusion, our study has shown: (i) a high proportion of pAmpC- and/or ESBL-producing clinical isolates of *E. coli* from the urinary tract and blood cultures at the Central Hospital [26]. CTX-Ms and FOX/MOX were the dominant ESBL- and pAmpC-types, respectively. (iii) All ESBL- and pAmpC-producing isolates were multidrug-resistant, only susceptible to antibiotics that are not easily available at the hospital. (iv) Studies of the genetic relatedness between ESBL- and / or pAmpC-producing isolates demonstrate genetic diversity and some clusters indicating within-hospital spread of isolates. The overall findings strongly support the urgent need for accurate and rapid diagnostic services to guide antibiotic treatment of life-threatening infections and improved infection control measures. The findings have a probable transfer value to other hospitals in Mozambique as the Central Hospital has reference functions with transfer of patients to and from other hospitals.

## Supplementary Information

**Additional file 1: Table S1.** Primer sequences for PCR amplification of ESBL and pAmpC genes. **Table S2.** Antimicrobial susceptibility for ESBL-positive *E. coli* blood isolates (*n* = 17). **Table S3.** Antimicrobial susceptibility for ESBL-positive *E. coli* urine isolates (*n* = 58). **Figure S1.** Cluster analysis of ESBL-positive *E. coli* (*n* = 75) isolates based on ERIC-PCR fingerprinting patterns using Jacquard index and UPGMA clustering. The scale at the top left represents percentage similarity. Isolate columns: identification with origins in brackets: O = outpatient, P = paediatrics, M = medicine, S = surgery, U = urine and B = blood. Presence (+) or absence (−) of *bla*_CTX-M_ and sequence type are also indicated for each strain.

## Data Availability

The datasets used and/or analysed during the current study are available from the first and the corresponding author on reasonable request.

## Abbrevations

AST: Antimicrobial susceptibility testing
ATCC: American Type Culture Collection
ERIC-PCR: Enterobacterial repetitive intergenic consensus PCR
ESBL: Extended-spectrum β-lactamase
ISCISA: Instituto Superior de Ciêncas de Saúde
MCH: Maputo Central Hospital
MDR: Multidrug-resistance
pAmpC: Plasmid-mediated AmpC β-lactamase
UKZN: University of KwaZulu-Natal
UPGMA: Unweighted Pair Group Method with Arithmetic Mean
