# Supplementary material for: High prevalence of multidrug resistant ESBL- and plasmid mediated AmpC-producing clinical isolates of Escherichia coli at Maputo Central Hospital, Mozambique
Source: BMC Infect Dis. 2021 Jan 6;21:16. doi: 10.1186/s12879-020-05696-y (PMC7789290; doi:10.1186/s12879-020-05696-y)
Supplement: Supplementary file 1 — Additional file 1: Table S1. Primer sequences for PCR amplification of ESBL and pAmpC genes. Table S2. Antimicrobial susceptibility for ESBL-positive E. coli blood isolates (n = 17). Table S3. Antimicrobial susceptibility for ESBL-positive E. coli urine isolates (n = 58). Figure S1. Cluster analysis of ESBL-positive E. coli (n = 75) isolates based on ERIC-PCR fingerprinting patterns using Jacquard index and UPGMA clustering. The scale at the top left represents percentage similarity. Isolate columns: identification with origins in brackets: O = outpatient, P = paediatrics, M = medicine, S = surgery, U = urine and B = blood. Presence (+) or absence (−) of blaCTX-M and sequence type are also indicated for each strain. [file 12879_2020_5696_MOESM1_ESM.docx]

**Table S1. Primer sequences for PCR amplification of ESBL and pAmpC genes**

| **Genes** | **Primers** | **Sequence( 5' to 3')** | **Annealing (ᵒC)** | **Reference** |
| --- | --- | --- | --- | --- |
| **TEM** | 1 | AAAATTCTTGAAGACG | 60 | [18] |
|  | 2 | TTACCAATGCTTAATCA |  |  |
| **CTX-M** | 1 | GGTTAAAAAATCACTGCGTC | 57 | [19] |
|  | 2 | TTGGTGACGATTTTAGCCGC |  |  |
| **SHV** | 1 | TTAACTCCCTGTTAGCCA | 56 | [18] |
|  | 2 | GATTTGCTGATTTCGCCC |  |  |
| **CMY** | 1 | GAT TCC TTG GAC TCT TCA G | 50 | [20] |
|  | 2 | TAA AAC CAG GTT CCC AGA TAG C | |  |
| **FOX** | 1 | CAC CAC GAG AAT AAC CAT | 50 | [20] |
|  | 2 | ATG TGG ACG CCT TGA ACT |  |  |
| **MOX** | 1 | GCTGCTCAAGGAGCA CAG GAT | 50 | [21] |
|  | 2 | CACATTGACATAGGT GTG GTGC | |  |
| **DHA** | 1 | AACTTTCACAGGTGTGCT GGGT | 50 | [21] |
|  | 2 | CCGTACGCATACTGGCTT TGC |  |  |

**Table S2. Antimicrobial susceptibility for ESBL-positive *E. coli* blood isolates (*n*=17)**

| **ID-number** | **Ward** | **CAZ** | **CTX** | **AMC** | **SXT** | **CIP** | **AMP** | **MER** | **GEN** | **FOX** | **PTZ** | **CRO** |
| --- | --- | --- | --- | --- | --- | --- | --- | --- | --- | --- | --- | --- |
| 1001 | Pediatric | R | R | R | R | S | R | S | S | S | S | R |
| 1010 | Medicine | R | R | S | R | S | R | S | S | S | S | R |
| 1181 | Pediatric | R | R | R | R | R | R | S | R | S | S | R |
| 1568 | Medicine | R | R | S | R | R | R | S | S | S | S | R |
| 2560 | Pediatric | R | R | I | R | S | R | S | R | S | R | R |
| 3507 | Medicine | R | R | S | R | S | R | S | S | S | S | R |
| 5848 | Medicine | R | R | R | R | R | R | S | S | S | R | R |
| 6389 | Medicine | R | R | R | R | R | R | S | S | S | S | R |
| 7017 | Pediatric | R | R | R | R | R | R | S | R | I | I | R |
| 8810 | Pediatric | R | R | R | R | S | R | S | R | R | R | R |
| 9344 | Pediatric | R | R | R | R | R | R | S | S | I | R | R |
| 12900 | Pediatric | R | R | R | R | R | R | S | S | I | I | R |
| 12968 | Medicine | R | R | R | R | R | R | S | R | S | R | R |
| 12999 | Pediatric | R | R | R | R | R | R | S | R | R | R | R |
| 13308 | Medicine | R | R | R | R | R | R | S | R | R | R | R |
| 13311 | Pediatric | R | R | R | R | R | R | S | S | S | S | R |
| 13313 | Medicine | R | R | R | R | R | R | S | R | R | R | R |

Abbreviations: S-susceptible; I-intermediate; R-resistant, CAZ-Ceftazidime; CTX-Cefotaxime; AMC-Amoxicillin-clavulanic acid; SXT-Trimethoprim-sulfamethoxazole; CIP-Ciprofloxacin; AMP-Ampicillin; MER-Meropenem; GEN-Gentamicin; FOX-Cefoxitin; PZT-Piperacillin-tazobactam; CRO-Ceftriaxone.

**Table S3. Antimicrobial susceptibility for ESBL-positive *E. coli* urine isolates (*n*=58)**

| **ID- NUMBER** | **Ward** | **CAZ** | **CTX** | **AMC** | **SXT** | **CIP** | **AMP** | **MER** | **NIT** | **GEN** | **FOX** | **PTZ** | **CRO** |
| --- | --- | --- | --- | --- | --- | --- | --- | --- | --- | --- | --- | --- | --- |
| 980 | Outpatient | R | R | R | R | I | R | S | S | S | S | R | R |
| 996 | Outpatient | R | R | R | S | S | R | S | S | R | S | R | R |
| 1000 | Outpatient | R | R | R | R | R | R | S | R | R | S | S | R |
| 1022 | Pediatric | R | I | R | R | I | R | S | S | R | S | S | R |
| 1037 | Pediatric | R | R | R | R | R | R | S | S | R | R | R | R |
| 1438 | Pediatric | R | R | R | R | S | R | S | S | R | S | S | R |
| 1482 | Medicine | R | R | R | R | S | R | S | S | S | I | S | R |
| 1620 | Medicine | R | R | R | R | R | R | S | R | S | R | I | R |
| 2596 | Pediatric | R | R | R | S | S | R | S | S | R | S | S | R |
| 3450 | Pediatric | R | R | I | R | S | R | S | S | R | S | S | R |
| 3463 | Pediatric | R | R | R | R | S | R | S | S | R | S | S | R |
| 3490 | Pediatric | R | I | R | S | S | R | S | S | S | I | R | R |
| 5917 | Pediatric | R | R | R | R | R | R | S | R | R | S | I | R |
| 4557 | Pediatric | R | R | R | R | S | R | S | S | S | I | S | R |
| 4980 | Medicine | R | R | R | R | S | R | S | S | S | R | S | R |
| 5100 | Pediatric | R | R | R | R | R | R | S | S | R | S | I | R |
| 5102 | Medicine | R | R | S | R | S | R | S | S | S | S | S | R |
| 5920 | Pediatric | R | R | R | R | R | R | S | R | S | R | I | R |
| 6002 | Pediatric | I | R | R | R | R | R | S | S | S | R | S | R |
| 6003 | Pediatric | R | R | R | R | R | R | S | R | R | S | R | R |
| 6057 | Pediatric | R | R | R | R | S | R | S | S | S | R | R | R |
| 6187 | Pediatric | R | R | R | R | R | R | S | S | S | S | I | R |
| 6514 | Pediatric | I | R | R | R | R | R | S | R | S | R | S | R |
| 6539 | Pediatric | R | R | R | R | R | R | S | S | R | S | R | R |
| 6670 | Outpatient | R | R | R | R | R | R | S | S | R | S | S | R |
| 6715 | Medicine | R | R | R | R | S | R | S | S | S | I | I | R |
| 6818 | Outpatient | R | R | R | R | R | R | S | R | R | S | S | S |
| 7421 | Pediatric | R | R | R | R | R | R | S | S | S | S | R | R |
| 7922 | Pediatric | R | R | R | R | R | R | S | I | S | S | R | R |
| 9479 | Medicine | R | R | R | R | R | R | S | S | S | R | R | R |
| 9481 | Pediatric | R | R | R | R | S | R | S | S | S | R | R | R |
| 10156 | Pediatric | R | R | R | R | R | R | S | S | R | S | R | R |
| 10642 | Outpatient | R | I | I | R | S | R | S | S | S | S | S | R |
| 10646 | Pediatric | R | R | R | R | R | R | S | S | S | R | S | R |
| 10822 | Outpatient | R | R | R | R | S | R | S | S | S | R | S | R |
| 10846 | Pediatric | R | R | R | R | S | R | S | S | R | R | R | R |
| 10864 | Pediatric | R | R | R | R | R | R | S | S | R | R | R | R |
| 10869 | Pediatric | R | R | R | R | R | R | S | S | S | R | R | R |
| 11460 | Pediatric | R | R | R | R | R | R | S | R | R | R | S | R |
| 11598 | Surgery | R | R | R | R | R | R | S | R | S | R | I | R |
| 12394 | Pediatric | R | R | R | R | S | R | S | R | S | S | S | R |
| 12958 | Pediatric | R | R | R | R | R | R | S | R | R | R | I | R |
| 13036 | Pediatric | R | R | R | R | R | R | S | S | R | R | S | R |
| 13088 | Medicine | R | R | R | R | R | R | S | I | R | R | R | R |
| 13040 | Pediatric | R | R | R | R | R | R | S | S | S | R | R | R |
| 13301 | Pediatric | R | R | R | R | R | R | S | R | R | R | S | R |
| 13302 | Pediatric | R | R | R | R | S | R | S | S | S | R | I | R |
| 13304 | Pediatric | R | R | R | R | R | R | S | R | S | I | R | R |
| 13305 | Pediatric | R | R | R | R | R | R | S | S | R | R | I | R |
| 13309 | Pediatric | R | R | R | R | R | R | S | R | I | R | R | R |
| 13310 | Pediatric | R | R | R | R | R | R | S | R | R | R | R | R |
| 13314 | Pediatric | R | R | R | R | R | R | S | R | S | R | R | R |
| 13315 | Medicine | R | R | R | R | R | R | S | R | S | R | R | R |
| 13316 | Medicine | R | R | R | R | R | R | S | R | S | S | S | R |
| 13320 | Surgery | R | R | R | R | R | R | S | R | S | R | R | R |
| 13335 | Pediatric | R | R | R | R | R | R | S | R | R | R | S | R |
| 13313 | Medicine | R | R | R | R | R | R | S | S | R | S | R | R |
| 10088 | Medicine | R | R | R | R | R | R | S | S | S | S | S | R |

Abbreviations: S-susceptible; I-intermediate; R-resistant; CAZ-Ceftazidime; CTX-Cefotaxime; AMC- Amoxicillin-clavulanic acid; SXT-Trimethoprim-sulfamethoxazole; CIP-Ciprofloxacin; AMP- Ampicillin; MER-Meropenem; NIT-Nitrofurantoin; GEN-Gentamicin; FOX-Cefoxitin; PTZ-Piperacillin-tazobactam; CRO-Ceftriaxone.

**Figure S1:** Cluster analysis of ESBL-positive *E. coli* (n=75) isolates based on ERIC-PCR fingerprinting patterns using Jacquard index and UPGMA clustering. The scale at the top left represents percentage similarity. Isolate columns: identification with origins in brackets: O = outpatient, P = paediatrics, M = medicine, S = surgery, U = urine and B= blood. Presence (+) or absence (-) of *bla*_CTX-M_ and sequence type are also indicated for each strain.
